# Fetal Immunomodulatory Environment Following Cartilage Injury—The Key to CARTILAGE Regeneration?

**DOI:** 10.3390/ijms222312969

**Published:** 2021-11-30

**Authors:** Iris Ribitsch, Andrea Bileck, Monika Egerbacher, Simone Gabner, Rupert L. Mayer, Lukas Janker, Christopher Gerner, Florien Jenner

**Affiliations:** 1 VETERM, Equine Surgery Unit, Department of Companion Animals and Horses, University of Veterinary Medicine Vienna, 1210 Vienna, Austria; Iris.Gerner@vetmeduni.ac.at; 2 Department of Analytical Chemistry, Faculty of Chemistry, University of Vienna, 1090 Vienna, Austria; Andrea.bileck@univie.ac.at (A.B.); Rupert.Mayer@univie.ac.at (R.L.M.); lukas.janker@univie.ac.at (L.J.); 3 Administrative Unit Veterinary Medicine, UMIT—Private University for Health Sciences, Medical Informatics and Technology GmbH, 6060 Hall in Tirol, Austria; monika.egerbacher@gmail.com; 4 Histology & Embryology, Department of Pathobiology, University of Veterinary Medicine, 1210 Vienna, Austria; simone.gabner@vetmeduni.ac.at

**Keywords:** cartilage, regeneration, inflammation, immunomodulation, macrophage, neutrophil, fetal

## Abstract

Fetal cartilage fully regenerates following injury, while in adult mammals cartilage injury leads to osteoarthritis (OA). Thus, in this study, we compared the in vivo injury response of fetal and adult ovine articular cartilage histologically and proteomically to identify key factors of fetal regeneration. In addition, we compared the secretome of fetal ovine mesenchymal stem cells (MSCs) in vitro with injured fetal cartilage to identify potential MSC-derived therapeutic factors. Cartilage injury caused massive cellular changes in the synovial membrane, with macrophages dominating the fetal, and neutrophils the adult, synovial cellular infiltrate. Correspondingly, proteomics revealed differential regulation of pro- and anti-inflammatory mediators and growth-factors between adult and fetal joints. Neutrophil-related proteins and acute phase proteins were the two major upregulated protein groups in adult compared to fetal cartilage following injury. In contrast, several immunomodulating proteins and growth factors were expressed significantly higher in the fetus than the adult. Comparison of the in vitro MSCs proteome with the in vivo fetal regenerative signature revealed shared upregulation of 17 proteins, suggesting their therapeutic potential. Biomimicry of the fetal paracrine signature to reprogram macrophages and modulate inflammation could be an important future research direction for developing novel therapeutics.

## 1. Introduction

Fetal cartilage fully regenerates following injury [1,2]. In contrast, adult cartilage has only negligible inherent reparative capacity but responds to injury by producing mechanically and functionally inferior fibrocartilage [3]. Cartilage injury in adult mammals, therefore, leads to chronic degenerative joint disease: osteoarthritis (OA) [3]. OA, the most common musculoskeletal disease affecting 240 million people globally, is associated with substantial morbidity, disability, reduced quality of life and commensurate socioeconomic costs [4,5,6,7,8,9]. Currently, no treatment is available to stop the progressive degeneration of articular cartilage in OA.

Although OA is characterized by a failure to repair damaged cartilage, it is a disease of the entire joint, affecting all articular tissues because of their physical and functional association [10]. The pathogenesis of OA is multifaceted, involving mechanical, cellular and molecular processes, inflammation, metabolic dysfunction and epigenetic modifications, and is orchestrated by intricate crosstalk including chondrocytes, synovial macrophages and fibroblasts, osteocytes and infiltrating leukocytes [11,12,13,14,15,16,17,18,19,20,21]. OA may occur as a result of a variety of predisposing factors, such as age and mechanical injury, that incite a cascade of pathophysiological events within articular tissues [11,12,13,14,15]. Cartilage ECM debris and intracellular alarmins released into the synovial microenvironment activate synovial macrophages, synovial fibroblasts, and chondrocytes to produce inflammatory and catabolic mediators, which in turn disrupt cellular homeostasis and the balance between matrix synthesis and degradation and thus create a vicious cycle of inflammation and cartilage breakdown [11,12,13,14,15].

The inflammatory response is amplified by an influx of leukocytes, predominantly macrophages but also neutrophils, natural killer cells, and a smaller number of T- and B-cell lymphocytes and mast cells from the vascular compartment [22,23,24,25,26,27,28]. Neutrophils are the first immune cells that accumulate in the joint (six hours), followed by macrophages (one day) and natural killer cells (three days) [28]. Much of the innate immune activation and cytokine production in the OA joint is attributed to synovial pro-inflammatory M1 macrophages, the key effectors of synovial inflammation that show significantly increasing numbers with increasing grade of inflammation [22,23,24,25,26,27]. In addition to their autocrine effects, paracrine interactions between macrophages and chondrocytes cause additional feedback loops and enhance synovitis and cartilage degradation [29]. The interplay between joint tissues and inflammatory mediators released by articular cells and infiltrating inflammatory cell populations contributes to the loss of articular homeostasis and disease progression [22,23,24,25,26,27]. Once inflammation is established, a dysregulated balance between pro- and anti-inflammatory polarized macrophages and the failure of synovial macrophages (Mɸ) to transition from M1 to M2 may contribute to the onset and progression of OA [30,31]. Interestingly, neutrophil depletion has been shown to ameliorate OA in a murine model [28], while macrophage depletion resulted in more equivocal results depending on the polarization of the macrophages and the selectivity of the depletion method; while CD14+ synovial macrophage depletion reduced cartilage degeneration, systemic and local depletion of colony-stimulating factor 1 receptor-expressing macrophages increased pro-inflammatory mediators and infiltration of monocytes and neutrophils into the joint synovium [30,32], indicating that macrophages are essential for immunomodulation.

Recently, we found differential regulation of inflammatory mediators in response to cartilage and tendon injury in fetal compared to adult sheep, suggesting a more robust immunomodulatory response in fetal animals [2]. Furthermore, in a comparison of fetal and adult tendon healing, we found a pronounced inflammatory response in adult injured tendons accompanied by activation of neutrophils and significant upregulation of pro-inflammatory factors and neutrophil-attracting chemokines. In contrast, in fetal regeneration, we observed prominent recruitment of macrophages with significant upregulation of anti-inflammatory proteins, several inflammation-resolving molecules and stem cell markers. Therefore, the present study aimed to identify key factors of fetal cartilage regeneration versus adult fibrocartilaginous repair and early OA development. To this end, shotgun proteomics and histologic analyses of tissue samples from fetal and adult injured compared to uninjured sheep were performed. The results were compared to the proteome profile of fetal MSCs to evaluate their therapeutic potential. We hypothesized that (a) the fetal response to cartilage injury is characterized by a rapid resolution of inflammation and the differential upregulation of immunomodulatory and growth factors, and (b) fetal MSCs in vitro respond to inflammatory stimuli by upregulating similar anti-inflammatory and pro-regenerative pathways as seen during the fetal injury response in vivo.

## 2. Results

### 2.1. Macroscopic and Histologic Evaluation of the In Vivo Injury Response

Upon harvest, 1- and 3-days (fetus) and 3-days (adult) post-injury (p.i.), the OARSI macroscopic score was 4/16 for cartilage because of the surgically-induced defect in the medial femoral condyle, and 0 for osteophytes and synovium. Fetal and adult control joints showed healthy articular cartilage with OARSI scores of 0 in all categories.

**Figure 1:**
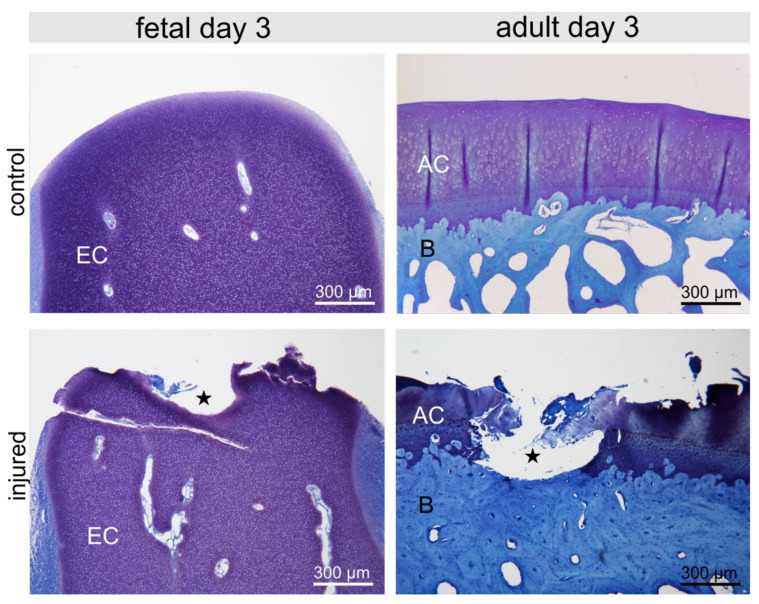
Fetal and adult control femoral condyle and condyle with lesion (asterisk) three days post-injury (p.i.) depicting Toluidine blue metachromatic staining of proteoglycans in the epiphyseal (EC) and articular cartilage (AC); bone (B).

The cartilage defect was clearly visible in fetal as well as in adult samples (Figure 1) without any morphologically visible repair attempt. Fetal and adult control condyles showed regular cartilage with a smooth articular surface (Figure 1).

**Figure 2:**
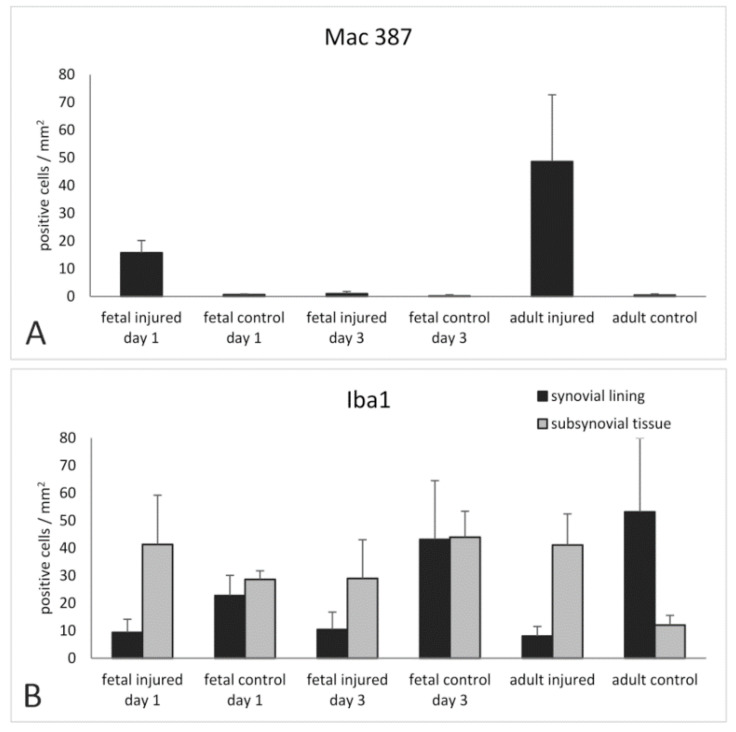
Mean numbers and standard deviation of Mac 387 (**A**) and Iba1 (**B**) positive cells per mm^2^ of synovial tissue.

**Figure 3:**
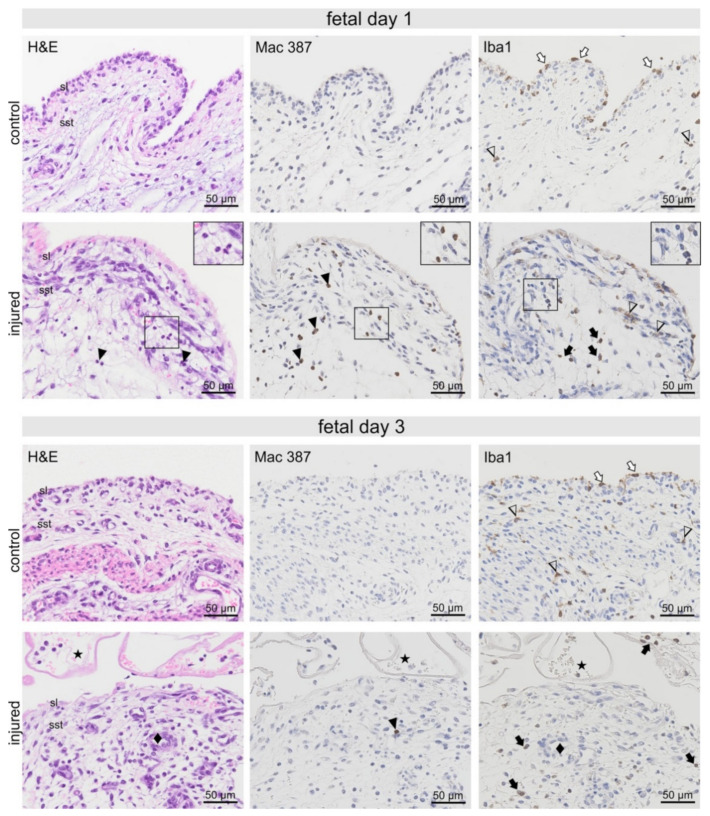
Fetal synovia from healthy and injured animals 1-day post-injury (p.i.). Nests of neutrophils (black arrowheads and inserts), which were readily detectable in H&E-stained sections, showed strong Mac 387 immunostaining but were negative for Iba1 (insert). Iba1-positive macrophages were prominent in the synovial lining (sl) of fetal control, but less obvious in the injured animal samples (white arrows). In the sub-synovial tissue (sst), irregularly shaped Iba1-positive cells (arrowheads) were found both in control and injured samples. However, additional round Iba1-positive macrophages (black arrows) were seen in samples of injured animals. Fetal synovia from healthy and injured animals 3-days post-injury (p.i.). Fibrin deposition and erythrocytes were observed on the synovial surface (asterisks). Striking blood vessels with high endothelium resembling HEV were seen (♦). Mac 387 positive neutrophils were mostly absent (black arrowhead). Macrophages seen in the synovial lining of control animals (white arrows) were almost absent in injured samples. Irregularly shaped Iba1-positive cells (arrowheads) were found in the control, and less prominent in injured sub-synovial tissue. Round Iba1 positive macrophages (black arrows) were abundant in the sub-synovial tissue and in the fibrin deposited on the synovial surface.

In fetal synovial samples 1-day p.i., affected samples could be identified by fibrin deposition on the synovial surface, extravascular erythrocytes, and synovial oedema. Nests of Mac 387-positive neutrophils, which were absent in control animals, were detected in the sub-synovial tissue 1-day p.i. (Figure 2 and Figure 3). Iba1-positive macrophages were prominent in the synovial lining of fetal control, but less obvious in the injured samples. In the sub-synovial tissue, irregularly shaped Iba1-positive cells were found both in control and injured samples. However, additional round Iba1-positive macrophages were seen in samples of injured animals (Figure 3). Extravascular erythrocytes and remnant fibrin deposition containing erythrocytes were still seen in fetal synovial samples three days p.i. (Figure 3). Only single Mac 387-positive neutrophils could be detected. In contrast to day 1 and control tissue, Iba1-positive macrophages in the synovial lining were almost absent in injured animals (Figure 2 and Figure 3). Irregularly shaped Iba1-positive cells were found in the control, and less prominent in injured sub-synovial tissue. Again, round Iba1 positive macrophages were most abundant in the sub-synovial tissue and in the fibrin deposited on the synovial surface.

**Figure 4:**
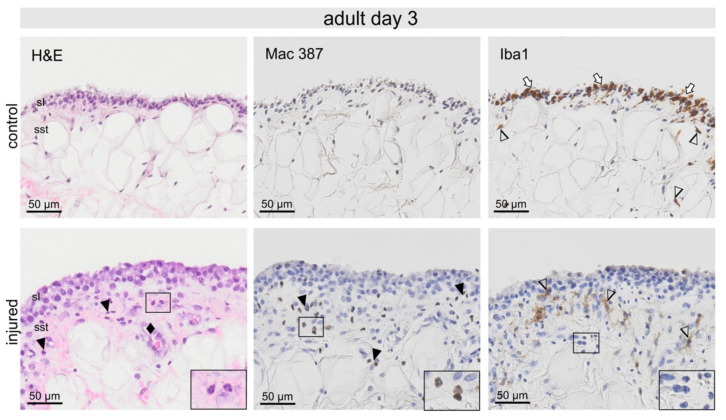
Adult synovia from healthy and injured animals 3-days post-injury (p.i.). Invasion of neutrophils detectable in H&E sections, showed strong Mac 387 immunostaining (black arrowheads and inserts), but were negative for Iba1 (insert). The synovial lining (sl) of control adult animals contained abundant Iba1-positive cells (white arrows); however, these cells were absent post-injury. In the sub-synovial tissue (sst), irregularly shaped Iba1-positive macrophages were accumulated directly below the synovial lining (arrowheads). Striking blood vessels with high endothelium resembling HEV were seen (♦).

In adult synovial samples 3-days p.i., fibrin deposition and extravascular erythrocytes were noticeable. The synovia appeared wider with a higher cell density when compared with the control samples (Figure 4). Invasion of neutrophils shown in H&E and Mac 387 staining into sub-synovial tissue was most prominent when compared with control samples (Figure 2 and Figure 4). The synovial lining of control adult animals contained more abundant Iba1-positive cells than in the fetus (Figure 2 and Figure 4). However, these cells were almost absent post-injury (Figure 2 and Figure 4). In the sub-synovial tissue, irregularly shaped Iba1-positive macrophages were accumulated directly below the synovial lining (Figure 4).

Both in fetal and adult injured synovial samples, the presence of high endothelial venules (HEV) was striking, indicating inflammatory cell recruitment (Figure 3 and Figure 4).

### 2.2. Mass Spectrometry of Adult and Fetal Cartilage Samples Following In Vivo Cartilage Injury

Out of a total of 2153 proteins identified in secretome fractions of cartilage samples (FDR < 0.01 for both protein and peptide levels, at least two peptides per protein), 57 proteins were found significantly upregulated upon injury in adults (more than two-fold change (FC), corrected for multiparameter analysis with an FDR < 0.05; individual *p*-values are indicated in Supplementary Table S1, whereas 67 proteins were found significantly down-regulated (FC < −2). As described previously, tissue-derived secretome samples contain not only genuinely secreted proteins but also proteins originating from cell debris, with some variance originating from the inherent complexity and heterogeneity of biological model systems and tissue samples [33,34]. Due to their functional implications, only genuinely secreted proteins significantly regulated between adult injured and adult control samples were included in the further analysis. Besides fifteen cell leakage proteins, the upregulated proteins comprised eighteen proteins related to neutrophil activation and neutrophil extracellular trap formation, ten acute phase proteins, four immunoglobulin-like proteins, two cytokines, five plasma proteins, one membrane protein and two ECM proteins. Among the downregulated proteins, 56 were found to be cell leakage proteins, in addition to three ECM proteins, three membrane proteins, one plasma protein, three platelet-derived proteins and one mucin.

**Figure 5:**
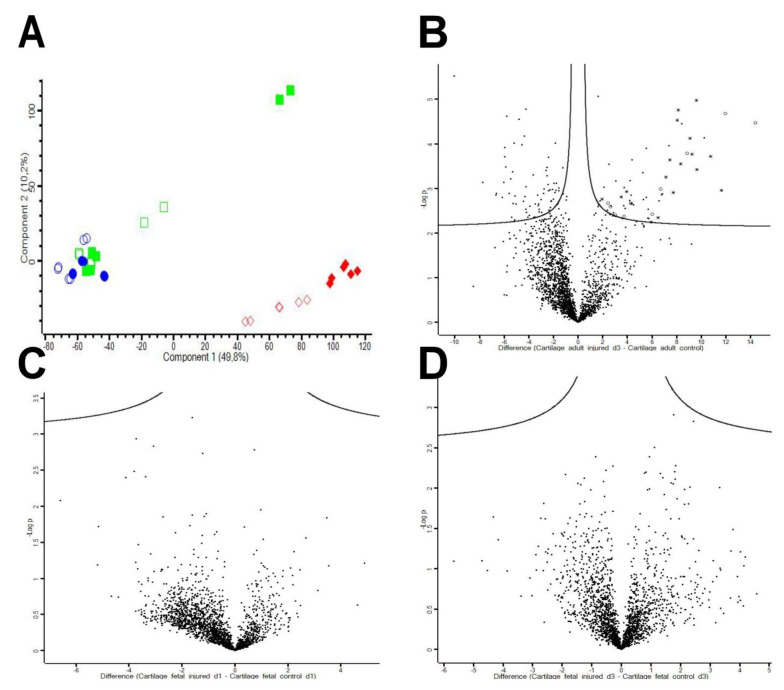
The fetal and adult injury response in vivo. (**A**) Principal component analysis (PCA) of adult and fetal secretomes derived from injured versus uninjured (control) cartilage samples (three biological and two technical replicates/group). PCA comparison shows clustering of adult injured and adult controls indicating a strong and robust difference in protein expression between adult uninjured control animals and animals 3-days post-injury. The fetal samples, although showing a less pronounced response to injury, cluster far away from the adult samples indicating a significant difference between fetal and adult samples. Adult control = red diamonds outlined; adult injured (day 3 post-injury (D3pi)) = red diamonds filled; fetal controls (D1pi) = green squares outlined; fetal injured (D1pi) = green squares filled; fetal control (D3pi) = blue circles outlined; fetal injured (D3pi) = blue circles filled). (**B**) Volcano plot showing the adult injury response. Three days post-injury, 57 proteins were significantly up-regulated, whereas 67 proteins were significantly down-regulated. The volcano plots indicate neutrophil (asterisk) and acute phase proteins (circles) in adults post-injury compared to the control samples. (**C**,**D**) Fetal injured sheep show little differences post-injury compared to uninjured controls (**C** = 1-day post-injury, **D** = 3-days post-injury).

**Figure 6:**
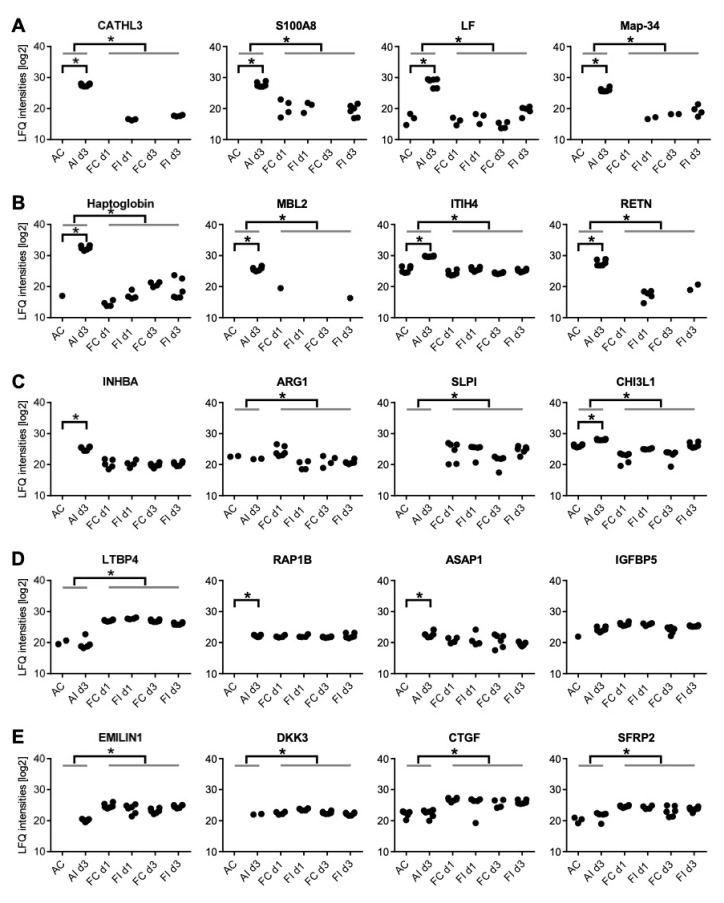
Differentially expressed proteins in adult and fetal sheep in response to injury. Comparison of adult and fetal secretomes from injured versus control cartilage (three biological and two technical replicates/group). (**A**) Neutrophil derived proteins—pointing to a higher neutrophil activity in adult than fetal samples. (**B**) Acute phase proteins showing a more pronounced upregulation in adult compared to fetal animals. (**C**) Macrophage marker proteins indicating a higher macrophage activity in fetal than adult samples. (**D**) Several growth factors with a high baseline expression in the fetus. (**E**) Proteins secreted by fetal MSCs which are described as healing-associated, and which were also detectable in the secretomes of the fetal in vivo samples and might thus be of therapeutic interest. AC: adult uninjured control samples; AId3: adult injured samples, 3-days post-injury; FCd1: fetal uninjured control samples, 1-day post-surgery of injured twins; FId1: fetal injured samples, 1-day post-injury; FCd3: fetal uninjured control samples, 3-days post-surgery of injured twins; FId3: fetal injured samples, 1-day post-injury; *: statistically significant difference, *p* < 0.05.

Remarkably, neutrophil-related proteins and acute phase proteins were the two major upregulated protein groups. While most of these proteins were also positively identified in fetal samples, the abundance levels were much lower compared to the adult samples and the contrast between injured and uninjured samples was far less pronounced (Figure 5 and Figure 6). However, although the difference between fetal injured and fetal control samples was not significant, proteins shown to be significantly upregulated in adults showed a consistent upregulation also in the fetus, suggesting that neutrophils and acute phase proteins were also involved in fetal regeneration.

### 2.3. Neutrophil-Derived Proteins Identified in the Secretome of Adult versus Fetal Ovine Cartilage 

Among the proteins significantly upregulated in adult sheep post-injury, lactotransferrin (LTF), cathelicidin 1-3 (CATL 1-3), S100 calcium binding protein A8 (S100A8) and myeloid antimicrobial peptide (Map-34) were particularly abundant (Figure 6A). Lactotransferrin is a marker for neutrophils and macrophages [35]. Cathelicidins are known as antimicrobial effector molecules characteristic for innate immune response and have been described in humans as biomarker for joint infection predominantly expressed by neutrophils [36]. Protein S100A8, contained in calprotectin, is reported to be a reliable blood marker for neutrophils [37]. Myeloid antimicrobial peptide (Map-34) is another effector molecule with anti-microbial properties, which was significantly upregulated in adult samples. Interestingly, this protein is described only for sheep and goats [38].

Other proteins which are characteristic for activated neutrophils and presently found to be significantly upregulated in adults but not in the fetus were high mobility group box 2 (HMGB2), histone cluster 1 H1 family member d (HIST1H1D), histones H2a, H3, H4 and H15 (LOC101106292, H2AFJ, H3F3A, LOC101109652), proteins S100 A9 and A12 (S100A9, S100A12), bactinecin (BAC11), neutrophil gelatinase-associated lipocalin (LCN2), H1 histone family member X (H1FX), and chitinase-3-like protein 1 (CHI3L1).

### 2.4. Acute Phase Proteins Identified in the Secretome of Adult versus Fetal Ovine Cartilage

Acute phase proteins are systemic inflammation markers induced by the action of pro-inflammatory cytokines and typically synthesized in the liver [39]. All acute phase proteins identified in our experiments were found upregulated in adult animals upon injury but only slightly in fetal animals, demonstrating a pronounced systemic inflammation in adults. Haptoglobin (HP) was reported to be found in arthritic synovial fluid, potentially forming pro-inflammatory fragments [40] (Figure 6B). Mannose binding lectin 2 (MBL2) is a pathogen recognition molecule, facilitating the opsonisation of pathogens and cell debris and potentially initiating the complement cascade [41]. Inter-alpha-trypsin inhibitor heavy chain family member 4 (ITIH4) is an anti-inflammatory type II acute phase protein, involved in inflammatory responses to trauma [42]. While RETN, which promotes chemotaxis of myeloid cells, is an adipokine mainly contributed by adipose tissue, its regulation resembles that of acute phase proteins [43].

Further potential acute phase proteins which were found significantly upregulated in adult sheep upon injury were serum amyloid A protein (SAA), serum amyloid A3.2 (SAA 3.2), mannan binding lectin serine peptidase 1 (MASP1), fibrinogen gamma chain (FGG), fibrinogen alpha chain (FGA) and a plasma proteinase inhibitor.

### 2.5. Macrophage Markers Identified in the Secretome of Adult versus Fetal Ovine Cartilage 

As suggested by our histological assessment, macrophage activity was significantly higher in fetal than adult cartilage (corrected for multiparameter analysis, FDR < 0.05), but showed no regulation upon injury (Figure 6C). Remarkably, both M1 (pro-inflammatory) and M2 (pro-regenerative) markers were detected: activin beta A (INHBA), which is preferentially released by M1 macrophages, promotes the pro-inflammatory phenotype and inhibits the formation of polarized M2 macrophages [44]. Arginase ARG1 is predominantly expressed by macrophages and represents a typical marker for M2 polarized macrophages [45]. Antileukoproteinase SLPI is an antibacterial protein with immunomodulatory and anti-inflammatory functions promoting M2 polarization, neutrophil apoptosis and clearance [46]. CHI3L1, a carbohydrate-binding lectin, is a marker for late stages of macrophage differentiation and takes part in macrophage bacterial killing but subsequently promotes M2 formation [47,48,49]. As expected, this protein was found induced also in adult animals upon injury, indicating that some immunomodulatory activity may also take place in the adults. The consistent expression of these marker molecules in fetal samples is a strong indicator for the presence of polarized macrophages in the fetal cartilage in contrast to adult tissue.

### 2.6. Anabolic Factors Identified in the Secretome of Adult versus Fetal Ovine Cartilage

In the fetus, several growth factors including Ras-related protein Rap1B, latent transforming growth factor beta binding protein 4 (LTBP4) and Arf-GAP with SH3 domain ANK repeat and PH domain-containing protein 1 (Asap1), have a high baseline expression potentially promoting a rapid anabolic response to injury (Figure 6D). Rap1B regulates crosstalk of signaling pathways during skeletal development by balancing the signaling effects of bone morphogenetic proteins and fibroblast growth factor during skeletal development and disease, and promotes chondrogenic fate and chondrogenesis [50]. LTBP4 is a key molecule required for the stability of the TGFβ receptor complex [51]. Similarly, Asap1, a regulator of cytoskeletal dynamics and biosynthetic trafficking, is essential for the morphogenesis of tissues derived from mesenchymal progenitor cells [52]. In contrast, in adults, these anabolic factors have a low baseline expression but are upregulated following injury.

### 2.7. Proteins Secreted by Fetal MSCs In Vitro, Which Were Also Identified in the Secretome of Adult versus Fetal Ovine Cartilage

Mesenchymal stem cells were reported to contribute to the fetal healing processes potentially via the secretion of proteins, which might thus be of therapeutic interest. In order to identify potentially interesting therapeutic candidate proteins, we searched for proteins secreted by fMSCs in vitro which are described as healing-associated, and which were also detectable in the secretomes of the fetal in vivo samples (Figure 6D,E, Table 1). Out of a total of 902 proteins identified in secretome fractions of fetal MSCs in vitro (FDR < 0.01 for both protein and peptide levels, at least two peptides per protein, Supplementary Table S2), 94 were considered as biologically relevant (genuinely secreted and known to be associated with wound healing/osteoarthritis based on literature research); of these, 17 were also highly expressed in injured fetal cartilage in vivo (Table 1). Among these, several candidate effector molecules potentially contributing to the healing processes, such as connective tissue growth factor (CTGF), dickkopf-3 (DKK3), secreted frizzled-related protein 2 (sFRP2), elastin microfibril interface (Emilin1), and insulin-like growth factor binding protein 5 (IGFBP5), could be discerned.

## 3. Discussion

The difference in regenerative capacity between fetal and adult tissues was accompanied by a significantly different histologic and proteomic response to injury. Fetal cartilage injury resulted in a rapid inflammatory response within one day, characterised by an infiltration of neutrophils into the sub-synovial tissue and upregulation of proinflammatory cytokines such as resistin (RETN) and cathelcidin-3 (CATHL3), demonstrating the fetal ability to mount an inflammatory response (Figure 6). By day 3 p.i., neutrophils had largely disappeared, and the fetal synovial immune cell population was dominated by Iba1-positive activated macrophages in the sub-synovial tissue (Figure 3).

The lack of differentially regulated proteins corroborated the histologically apparent rapid resolution of inflammation in fetal injured joints. In contrast, an equivalent injury to adult cartilage yielded a significantly stronger inflammatory response with 124 differentially regulated proteins at day 3 p.i., of which 18 were proteins characteristic for neutrophils, such as CATHL3, S100A8 and lactotransferrin. In addition, acute phase proteins including haptoglobin (HP), RETN, mannose-binding lectin 2 (MBL2) and inter-alpha-trypsin inhibitor heavy chain H4 (ITIH4) were significantly higher regulated in adult injury. Indeed, cathelicidin and S100A 8 levels have been shown to correlate with cellular influx in collagen-induced arthritis, demonstrating their fundamental role in OA pathogenesis and the perpetuation of inflammation [53]. Additionally, synovial haptoglobin levels strongly correlate with the severity of OA [54]. Similarly, lactotransferrin, a known neutrophil activation marker protein, and resistin, which has been shown to enhance neutrophil extracellular trap (NET) formation, indicate high neutrophil activation and are associated with synovitis and sustained inflammation [55]. Histology further confirmed neutrophils as the primary immune cell in the adult synovial tissue.

Innate immune cell activation, particularly neutrophil function, has a profound role in OA pathology and, while critical to the injury response, can also cause extensive collateral tissue damage [56]. Neutrophils induce rapid damage to articular cartilage and degrade cartilage proteoglycan up to 28 times more than either macrophages or synovial fibroblasts [57]. Correspondingly, neutrophil depletion has been shown to ameliorate OA in a murine model and preserve the joint architecture, reduce cell infiltration and synovitis, chondrocyte apoptosis and glycosaminoglycan loss [28]. Since the elimination of inflammatory neutrophils from the site of inflammation is a prerequisite for resolution of the acute inflammatory response [58], the rapid clearance of neutrophils from the synovial tissue in fetal joint injury as shown in the current study may contribute to the efficient fetal resolution response.

The role of Mɸ in the context of OA pathogenesis is more diverse and dependent on their polarization state that dynamically responds to microenvironmental cues [30,59,60]. On the one hand, dysregulation of Mɸ activation with non-resolving persistent macrophage-mediated synovial inflammation is considered as one of the main drivers of both the establishment and progression of OA [30,31,61]. Indeed, the accumulation of M1 polarized macrophages in the intimal lining is the principal morphological characteristic of synovitis and correlates with the progression of OA, stiffness, function and quality of life [22,29,62,63,64]. On the other hand, macrophages are critical immunomodulators in joint injury and have a pivotal role in reinstating articular homeostasis and functional healing [30,32,60,62,65,66,67]. Corresponding to their diverse functional spectrum, macrophage depletion studies yielded equivocal effects depending on the targeted subpopulations, exacerbating rather than diminishing disease [30,32,62,68,69]. These studies indicate that the failure to transform from the M1 to M2 subtype may play a larger role in the progression of OA than the quantity of activated macrophages [29]. This was confirmed in transgenic mouse models, which showed that synovial macrophage M1 polarization exacerbated experimental collagenase-induced OA, while M2 polarization attenuated OA development [70]. Interestingly, in the present study, Iba1^+^ (“pan-macrophage marker”, ref. [71]) synovial lining macrophages disappeared at day 3 p.i. in both adult and fetal joints while Iba1+ cells appeared in the sub-synovial tissue, indicating either migration into the sub-synovial tissue or loss into the synovial fluid or both. However, in the fetal samples, the presence of round Iba1+ macrophages in the sub-synovia was more prominent, potentially indicating recruitment of monocytes from the circulation.

While the crucial role of macrophages in morphogenesis is well-established [72,73,74,75], the upregulation of macrophage marker proteins such as scavenger receptor cysteine-rich domain-containing protein (SSC5D) and secretory leucocyte protease inhibitor (SLPI) in both control and injured fetal animals was unexpected but may contribute to the rapid switch from a neutrophil to a macrophage dominated injury response and the efficient resolution of inflammation. SLPI promotes neutrophil apoptosis and their subsequent clearance, and reprograms myeloid cells towards resolution responses [46]. Similar to the fetus, adult cartilage also upregulated macrophage markers, such as the M2 macrophage marker arginase1 (Arg1) and chitinase-3-like protein 1 (CHI3L1) [49,76], in response to injury. However, in contrast to the fetus, the adult attempt to modulate the inflammatory response toward resolution seems to fail.

Like all biological cascades, the inflammatory response is shaped by a delicate balance between positive and negative feedback loops; moreover, the resolution of inflammation is an active process rather than a passive return to homeostasis [77,78]. Events at the onset of acute inflammation already establish biosynthetic circuits for a series of chemical mediators that later not only antagonistically inhibit the inflammatory cascade but also agonistically actively promote resolution [77]. Anti-inflammation and pro-resolution are therefore not equivalent but fundamentally different at both the molecular and cellular levels [77]. As in joint injury, the innate immune response also encodes part of the stromal cell repertoire’s response towards cell stress and damage, implicating chondrocytes and synovial fibroblasts in addition to the classical immune effector cells; immunomodulatory strategies must address the articular homeostasis and not selectively the immune system to successfully resolve the inflammatory response [69,79]. Transition from the inflammatory to the proliferative phase is a crucial step of the healing process and accumulating evidence associates a compromised transition with fibrosis and scarring [80]. Thus, targeting factors that impact this phase transition may offer a novel therapeutic strategy [80].

Furthermore, several growth factors were shown to have a high baseline expression, potentially promoting a rapid anabolic response to injury in the fetus, while in the adult these anabolic factors are upregulated following injury after the catabolic processes induced by pro-inflammatory cytokines have already dysbalanced the articular homeostasis. Although adult injured cartilage upregulates many factors also seen in fetal animals, the timing and magnitude of the response seem insufficient to achieve regeneration. Interestingly, several factors associated with OA, such as SSC5D and periostin [81,82], are constitutively expressed in fetal animals, indicating that these factors are not associated with pathogenesis per se but rather with morphogenetic or reparative processes. Consequently, therapeutic strategies need to modulate the adult articular microenvironment to facilitate successful repair.

In addition to the immune system, chondrocytes and articular MSC populations are major contributors to the healing response. Joint resident MSCs and progenitor cells, contrary to longstanding historical beliefs, are actually relatively abundant in vivo even in adults and occupy several articular niches, including the superficial cartilage zone, synovium and synovial fluid [83,84,85,86,87,88,89,90,91]. Interestingly, several cell tracing studies showed that MSCs found in experimental cartilage defects in vivo predominantly originate in the synovial membrane rather than in the superficial zone [86,89,92]. MSC senescence and an associated loss of potency could be an important facet of OA pathophysiology [93,94,95]. Indeed, advanced OA is associated with a numerical increase, but functional decline, in MSCs; while on the other hand, osteoarthritic synovium impacts chondrogenic differentiation of MSCs via macrophage polarisation state, thus closing the vicious circle of OA cellular crosstalk [91,93,96,97]. In contrast, fetal stem cells such as umbilical cord-derived MSC have superior anti-inflammatory and immunosuppressive properties compared to MSCs from adult tissues [98].

To assess the potential of fetal MSCs secretome as novel regenerative treatment, we also examined the secretory response of fetal MSCs in vitro to inflammatory stimuli and found several growth factors, such as CTGF, DKK3, Emilin1, periostin, sFRP2 and IGFBP5, highly expressed in the fetus, which were also expressed by fetal MSCs in vitro, suggesting a potential therapeutic role. Indeed, CTGF plays critical roles in cartilage development, maintenance, and regeneration [99,100]. Dkk3 has a well-established protective effect on cartilage integrity by preventing proteoglycan loss, modulating inflammatory cell activity and helping to restore OA-relevant signaling pathway activity [101,102]. In addition, DKK3 was described to regulate functional differentiation of various forms of stem cells including in smooth muscle or intestinal crypts [103,104]. Emilin1 was described as a marker molecule for regenerative multipotent mesenchymal stromal cell promoting the regeneration of damaged pancreatic islets [105]. In our samples, Emilin1 was found up-regulated in adults upon injury but was expressed at significantly higher levels in fetal tissue samples. Periostin, a regulator of the Wnt/beta-catenin signaling pathway, was recently described to promote functional regeneration and repair of tendons [106]. SFRP2 has recently been highlighted as a key molecule for the biogenesis of a regenerative phenotype in MSCs [107,108]. Similarly, insulin-like growth factor binding proteins such as IGFBP5 have been recently described to modulate matrix synthesis in degenerated areas of osteoarthritic cartilage in a healing-promoting fashion [109].

One inherent limitation of the in vivo comparison of fetal and adult cartilage healing lies in the difference in articular cellularity and vascularity. In this study, we tried to compensate for the latter by creating microfractures in adult cartilage defects to access the bone marrow. The difference in chondrocyte cellularity, which decreases at the articular surface between fetal and adult cartilage from 290 to 150 million cells per cm^3^ and the corresponding distance to the nearest neighbouring cell, which increases with maturity from 7.9 microns to 9.1 microns [110], remains a confounding factor, which must be taken into account in the development of biomimetic therapeutic strategies.

Furthermore, while no in vitro model can fully emulate the in vivo situation, we tried to address the inherent limitations of in vitro studies and to closely mimic the in vivo stimulation of these cells by using the secretome of inflamed chondrocytes to precondition the MSCs and IL1 and TNFa, key inflammatory stimuli in OA, to induce inflammation in chondrocytes.

As OA is one of the most common diseases among mammals and the world’s leading cause of chronic disability not only for the elderly but also for individuals of working age [111], novel disease-modifying treatments are urgently needed. Given the crucial role of inflammation in OA, inducers/agonists of the resolution phase of inflammation have enormous therapeutic potential [77]. Indeed, the rapid fetal resolution of inflammation appears to be key for a successful response to injury. Biomimicry of the fetal paracrine signature to reprogram macrophages and modulate inflammation could thus be an important future research direction for developing novel therapeutics for OA. In fact, paracrine factors secreted by fetal MSCs in vitro show substantial parallels to the in vivo regenerative signature, suggesting their therapeutic potential.

## 4. Materials and Methods

### 4.1. Experimental Design

To investigate the fetal immunomodulatory environment, the fetal and adult in vivo response to cartilage injury was assessed in an ovine medial femoral condylar defect model and compared using comprehensive histological and proteomic analyses, with the latter focusing on neutrophil, macrophage and stem cell markers, and acute phase proteins. In addition, proteins expressed in fetal inflamed MSCs in vitro were compared with the in vivo response to injury to identify potential MSC-derived therapeutic factors.

### 4.2. In Vivo Cartilage Injury Model

Standardized cartilage lesions were created (ethical approval numbers 68.205/0155-WF-/V/3b/2014 and 68.205/0028-II/3b/2014) in musculoskeletally mature (2–5 years, body weight 95 ± 12 kg), female Merino-cross ewes (*Ovis aries*) without orthopaedic disease, and fetal lambs (80 gd, term = ∼150 days) as previously described [2]. Day 80 of gestation was chosen to be able to study the regenerative response in the presence of a fully functioning immune system and established ability to mount an inflammatory response, which fetal sheep acquire by gestational day 65–75 [112,113,114,115,116]. For the fetal subjects, only twin pregnancies were included to provide a twin lamb as an uninjured control to allow differentiation between protein secretion of normal fetal development and fetal response to cartilage injury.

Following a minimally invasive medial parapatellar arthrotomy [117], bilateral full-thickness cartilage lesions with a diameter of 7 mm (adult sheep) or 1 mm (fetal lamb) and a depth of 1 mm were created in the medial femoral condyle. To ensure that differences in the healing response between fetal and adult articular cartilage are not caused by differences in blood supply due to fetal cartilage channels [1], we generated three microfractures of standardized size, number and distribution in adult cartilage defects to access the bone marrow vasculature, as evidenced by visible bleeding in all defects [118]. Adult animals were allowed full weight-bearing immediately after surgery. Pain management was provided with morphine.

Tissue samples were harvested immediately following euthanasia on day one and three (fetal twin sheep, injured and control), day 0 (uninjured control) and day three after injury (adult sheep), respectively, from three biological replicates per comparison group. After macroscopic evaluation using the OARSI scoring system [119], the medial femoral condyles and patellae with attached synovial membrane were surgically excised, and left and right knees were randomly assigned to mass spectrometry and histology. For mass spectrometry, the (cartilage) tissue remnants contained in the defect area and the cartilage rim surrounding the lesion (3 mm width in adults; 1 mm width in fetal sheep) were excised, cultivated in serum-free RPMI medium (Gibco, Life Technologies, Vienna, Austria) supplemented with 1% penicillin and 1% streptomycin (ATCC, LGC Standards GmbH, Wesel, Germany) for 6 h, sterile-filtered (0.2 µm filter), precipitated with ice-cold ethanol and stored at −20 °C until further processing. In addition, adult articular cartilage and umbilical cord blood was harvested for chondrocyte and MSC isolation.

### 4.3. Cell Isolation and Culture 

To determine the proteomic fingerprint of fetal MSCs in vitro for comparison with the in vivo secretome, MSCs were isolated from umbilical cord blood of fetal sheep (n = 3) as previously described [120,121]. In brief, following standard density gradient centrifugation (400 *g* at 20 °C for 30 min) using a polysaccharide solution (Ficoll-Paque Premium, GE Healthcare, Vienna, Austria), mononuclear cells were resuspended in standard culture medium consisting of DMEM (low glucose, without L-Glutamine (Lonza, Visp, Valis, Switzerland), 10% FCS (low endotoxin (Sigma Aldrich, St. Louis, MO, USA), 1% L-Glutamine (L-Alanyl L-Glutamine 200 mM, Biochrom, M&B Stricker, Bernried, Bavaria, Germany), 1% Pen/Strep (100×, Sigma Aldrich) and 1% Amphotericin B (250 µg/mL, Biochrom, M&B Stricker, Bernried, Bavaria, Germany), seeded on a culture dish and cultured in a humidified atmosphere at 37 °C and 5% CO_2_. The following day, the cells were washed to remove non-adherent cells and tissue debris. MSCs were characterized using FACS and trilineage differentiation as previously described (details see Supplementary material S3 and Supplementary Figure S4) [120,122].

For biomimicry of the in vivo inflammatory stimulation of MSCs (preconditioning of MSCs), adult articular cartilage was harvested and chondrocytes isolated as previously described [121]. In brief, articular cartilage samples were minced into small pieces (1 mm^3^), digested in a 1 mg/mL collagenase (Type I, Sigma Aldrich) solution for 6–8 h under constant stirring at 37 °C, and filtered through a cell strainer (100 µm, Greiner Bio One, Kremsmünster, OÖ, Austria). The obtained chondrocytes were washed twice (DPBS + Ca/Mg (Lonza; centrifugation at 400× *g* for 5 min at room temperature). The resulting cell pellet was resuspended in standard culture medium (equivalent to the MSCs). Chondrocytes were seeded onto a culture dish and cultured in a humidified atmosphere at 37 °C and 5% CO_2_. The following day, the cells were washed to remove non-adherent cells and tissue debris.

Medium was changed twice weekly. Passaging was performed upon colony confluence for the first passage and later upon 80–90% confluence by trypsinisation (Trypsin 0.05%, EDTA 0.02%, Biochrom, M&B Stricker, Bernried, Bavaria, Germany). MSCs and chondrocytes were frozen and stored in liquid nitrogen until further use at the lowest possible passage (P1-2).

### 4.4. Histology and Immunohistochemistry

For histological analysis, the femoral condyles and synovial membranes (n = 3/group) were fixed in 4% buffered formalin and processed as previously described [2,123,124]. Condyles from adult sheep were decalcified in 8% neutral EDTA. After routine embedding in paraffin, 4-μm-thick sections were mounted on APES-glutaraldehyde-coated slides (Sigma-Aldrich, St. Louis, MO, USA). Consecutive sections were stained with hematoxylin and eosin (H&E), Toluidine blue, and Safranin-O, and evaluated based on the International Cartilage Repair Society (ICRS) criteria [125,126], including the cartilage surface and matrix, cell distribution, cell viability and subchondral bone, but without scores as no defect repair can occur in a 3-day time frame. Consecutive sections of synovial membranes were stained with H&E or immunohistochemically.

For immunohistochemical detection of neutrophils (via mouse monoclonal antibody Macrophage/Calprotectin Ab-1 [Mac 387], Thermo Fisher Scientific, Waltham, MA, USA) and macrophages (via rabbit polyclonal antibody Ionized calcium binding adaptor molecule 1 [Iba1], Wako, Neuss, Germany), sections were deparaffinised, rehydrated, and endogenous peroxidase was blocked with 0.6% hydrogen peroxide in methanol (15 min at room temperature). Sections were pre-treated with 1 mg/mL protease (Sigma Aldrich, P5147; 20 min at room temperature) for Mac 387 staining, and 0.01 M citrate buffer (pH 6.0, 2 h in a water bath at 65 °C) for Iba1 staining. Nonspecific binding of antibodies was prevented by incubation with 1.5% normal goat serum (Dako Cytomation, Glostrup, Denmark) in phosphate-buffered saline (PBS; 30 min at room temperature). Primary antibodies were diluted in PBS (1:2000 for Mac 387, and 1:4000 for Iba1) and incubated overnight at 4 °C. An appropriate BrightVision Peroxidase system (Immunologic, Duiven, The Netherlands) was used and peroxidase activities were localized with diaminobenzidine (DAB Quanto Chromogen TA-125-QHDX, Thermo Fisher Scientific). Cell nuclei were counterstained with Mayer’s hematoxylin.

Tissue from sheep lymph node (Mac 387) and sheep liver (Iba1) served as positive controls. For negative control experiments, the primary antibody was omitted. For quantification, Mac 387 and Iba1 positive cells were counted and the synovial tissue area measured in five regions of interest (ROI), each at 20× magnification. Afterwards, the numbers of positive cells per mm^2^ of synovial tissue were calculated.

### 4.5. Fetal MSCs Secretome Production

Fetal ovine umbilical cord blood derived MSC and adult ovine articular chondrocytes (n = 3 biological and n = 2 technical replicates per cell type) were thawed and cultured (in T175 cell culture flasks, Sarstedt, Nümbrecht, Germany) in standard culture medium to passage 2 to 4, then cells were transferred to StemMACS™ MSC Expansion Media (Miltenyi Biotec, Bergisch Gladbach, Germany) plus corresponding medium supplements as per the manufacturer’s instructions.

For inflammatory preconditioning of the fetal MSCs, adult chondrocytes (100,000 cells/well in 12-well-plates, Sarstedt) were chemically inflamed by adding 10 ng/mL IL1β and TNFα (Immuno Tools, Friesoythe, Germany) to StemMACS^TM^ medium for 24 h. Thereafter, the medium was changed to StemMACS^TM^ without inflammatory stimuli and chondrocyte supernatants were harvested after 6 h incubation. Fetal MSCs (100000 cells/well in 12-well-plates) were cultured in the supernatant of the inflamed chondrocytes for 24 h to simulate naturally inflammatory stimuli occurring in the joint. Thereafter, the medium was changed to StemMACS^TM^ medium with only 0.14% of the provided medium supplements (to minimize cell death; higher quantities were not possible as the proteins contained in the supplement would overshadow the mass spectrometry measurement results). Fetal MSC secretomes were harvested after 6 h incubation and centrifuged at 4° at 1400× *g* for 5 min. The obtained supernatant was precipitated in ice cold 99.6% ethanol and stored at −20 °C until further processing and downstream analysis.

### 4.6. Mass Spectrometry 

After precipitation, proteins were dissolved in sample buffer (7.5 M urea, 1.5 M thiourea, 4% CHAPS, 0.05% SDS, 100 mM dithiothreitol (DDT)) and protein concentrations were determined using Bradford assay (Bio-Rad Laboratories, Munich, Germany).

A 20 μg sample of each protein was used for a filter-aided digestion, as described previously [127,128,129,130,131]. Briefly, 3 kDa molecular weight cut-off filters (Pall Austria Filter GmbH) were conditioned with MS-grade water (Millipore GmbH). Protein samples were concentrated on the pre-washed filter by centrifugation at 15,000× *g* for 15 min. After reduction with DTT [5 mg/mL dissolved in 8 M guanidinium hydrochloride in 50 mM ammonium bicarbonate (ABC) buffer, pH 8] and alkylation with iodoacetamide (10 mg/mL in 8 M guanidinium hydrochloride in 50 mM ABC buffer), samples were washed and 1 µg trypsin was added before incubation at 37 °C for 18 h. After enzymatic digestion, peptide samples were cleaned with C18 spin columns (Pierce, Thermo Scientific, Germany), dried, and stored at −20 °C until analysis.

For mass spectrometric analyses, dried samples were reconstituted in 5 µL 30% formic acid (FA) containing 10 fmol of each of the four synthetic standard peptides and diluted with 40 µL mobile phase A (H2O:ACN:FA = 97.9:2:0.1). A 10 µL aliquot of the peptide solution was loaded onto a 2 cm × 75 µm C18 Pepmap100 precolumn (Thermo Fisher Scientific) at a flow rate of 10 µL/min using mobile phase A. Afterwards, peptides were eluted from the precolumn to a 50 cm × 75 µm Pepmap100 analytical column (Thermo Fisher Scientific, Waltham, MA, USA) at a flow rate of 300 nl/min, and separation was achieved using a gradient of 8% to 40% mobile phase B (ACN:H2O:FA = 79.9:20:0.1) over 95 min. For mass spectrometric analyses, MS scans were performed in the range of m/z 400–1400 at a resolution of 70,000 (at m/z = 200). MS/MS scans of the eight most abundant ions were achieved through high-energy collisional dissociation fragmentation at 30% normalized collision energy and analysed in the orbitrap at a resolution of 17,500 (at m/z = 200). All samples were analysed in duplicate. Untargeted proteome profiling of secreted protein fractions derived from healthy and injured sheep cartilage tissue as well as fetal MSCs was performed, extending our previously published dataset, which established the feasibility of our fetal cartilage injury model [2].

### 4.7. Data Analysis 

Protein identification and label-free quantitative (LFQ) data analysis were performed using the open source software MaxQuant (version 1.3.0.5, Max Planck Institute of Biochemistry, Martinsried, Germany) including the Andromeda search engine [132]. Protein identification was achieved searching against *Ovis aries* in the Uniprot Database (version 09/2014 with 26,864 entries), allowing a mass tolerance of 5 ppm for MS spectra and 20 ppm for MS/MS spectra, as well as a maximum of two missed cleavages. In addition, carbamidomethylation on cysteine residues was included as a fixed modification, whereas methionine oxidation and N-terminal protein acetylation were included as variable modifications. Furthermore, search criteria included a minimum of two peptide identifications per protein, at least one of them unique, and the false discovery rate (FDR) calculation based on q-values, performed for both peptide identification as well as protein identification, less than 0.01. Prior to statistical analyses, proteins were filtered for reversed sequences, contaminants, and a minimum of three independent identifications per protein. Proteins identified in the present work were assigned to human orthologues and subsequently classified according to cell leakage, or genuinely secreted based on the UniProt database (accessed February–May 2021). Statistical evaluation was performed with Perseus software (version 1.6.0.2) using LFQ intensities of the MaxQuant result file [133]. After filtering potential contaminants, the LFQ values were log(2)-transformed. Technical duplicates were averaged. Only proteins detected in three of three biological replicates in either control and/or treatment groups were considered for data evaluation. Permutation-based FDR was set to 0.05 for *t*-tests and provided significant protein expression changes corrected for multi-parameters (S0 = 0.1).

## 5. Conclusions

In summary, the innate immune system may cause substantial tissue damage in adults due to molecular dysregulation and persistent inflammation. Defence against potential pathogens orchestrated by neutrophils appears to be evolutionarily prioritized over regenerative processes in the adult. In the fetus in contrast, there seems to be a concerted effort to limit innate immune system-induced cell damage supported by higher macrophage activity, which accelerates clearance of debris and resolution of inflammation, considered to be a crucial prerequisite for successful regeneration.

## Figures and Tables

**Table 1:** List of 17 proteins, which are highly expressed both in injured fetal cartilage in vivo and in fetal inflamed MSCs in vitro. Calculated q-value regarding protein identification confidence was 0 for all listed proteins.

Protein Name	Gene Name	UniProt Accession Number	Max. Quant. Score	Intensity	MS/MS Count
Connective tissue growth factor	CTGF	C7EDS5	80.116	4,281,300,000	241
Dickkopf-3	DKK3	W5PHV5	31.057	224,210,000	33
Elastin microfibril interface	EMILIN1	W5QCQ3	8.1415	39,950,000	15
Fibrillin-2	FBN2	W5Q6X0	184.29	572,190,000	91
Fibulin-5	FBLN5	W5PSC8	29.544	867,790,000	88
Inhibin beta a	INHBA	W5Q7R4	8.3799	179,290,000	17
Insulin-like growth factor binding protein 5	IGFBP5	B3GS77	108.13	2,128,300,000	190
Nidogen-1	NID1	W5P094	101.44	656,120,000	102
Nidogen-2	NID2	W5QHQ8	101.56	1,151,100,000	163
Olfactomedin-like protein 2B	OLFML2B	W5PLG3	8.0108	59,673,000	13
Periostin	POSTN	W5PK03	323.31	1.3×10^10^	870
Peroxidasin	PXDN	W5P5G3	6.927	95,649,000	12
Platelet-derived growth factor receptor-like protein	PDGFRL	W5PHH5	36.858	980,960,000	81
Ras-related protein	Rap1b	W5NSD5	57.332	3,110,100,000	364
Secreted frizzled-related protein 2	sFRP2	C5IWT3	84.427	1,302,800,000	111
SERPIN domain-containing protein	SERPINF2	W5PXU6	97.203	1,648,900,000	191
Transforming growth factor-beta-induced protein ig-h3	TGFBI	W5Q0F3	323.31	2.0637×10^10^	612

## Data Availability

The datasets generated and analyzed during the current study are available from the corresponding authors on reasonable request. The mass spectrometry proteomics data have been deposited to the ProteomeXchange Consortium via the PRIDE partner repository [134] with the dataset identifier PXD027486.

## Supplementary Materials

The following are available online at https://www.mdpi.com/article/10.3390/ijms222312969/s1.
