# Supplementary material for: Fetal Immunomodulatory Environment Following Cartilage Injury—The Key to CARTILAGE Regeneration?
Source: Int J Mol Sci. 2021 Nov 30;22(23):12969. doi: 10.3390/ijms222312969 (PMC8657887; doi:10.3390/ijms222312969)
Supplement: Supplementary file 1 [file ijms-22-12969-s001.zip › S3_Supplementary_Material.pdf]

Supplementary Material

1 Cell characterization: Methodology

Flow Cytometry

Cell surface antigen expression of MSCs was analysed using positive and negative surface markers to define their stem cell character (Dominici 2006). For flow cytometry, cells at passage 2 were detached from the flask using trypsin. MSC were washed with FACS buffer containing PBS, 3% FCS and 0.1% NaN_3 (Sigma Aldrich) and pelleted by centrifugation for 5 min at 400g. 1 x 10^5 cells per sample were incubated with a specific primary antibody or an isotype-matched control at 4ºC. After 30 minutes, unbound antibody was removed by washing with 1ml FACS buffer. The cell pellets were resuspended in 200µl FACS buffer and analysed with a BD FACSCanto II (BD Biosciences, Franklin Lakes, New Jersey, USA). 1 x 10^4 events were recorded per sample. The results were analysed by FlowJo (10.0.7) software (FlowJo LLC, Ashland, Oregon, USA).

MSC Trilineage Differentiation

MSCs (passage 2-4) were differentiated towards the three mesenchymal lineages: Cartilage, bone and fat (Dominici 2006). For that purpose, they were maintained in differentiation medium (StemPro® Chondrogenesis Differentiation Kit, StemPro® Osteogenesis Differentiation Kit and StemPro® Adipogenesis Differentiation Kit, Gibco, Fisher Scientific, Waltham, MA, USA) with media changes twice weekly, for 21 days. MSCs cultured for the same time period but in standard culture medium served as control.

Chondrogenic differentiation was performed in pellet culture, osteogenic and adipogenic differentiation in monolayer. However, osteogenically differentiated cells formed nodules within the 21 day culture period, which were further processed like a pellet culture. All samples were fixed with 4% formalin (ACM, Herba Chemosan Apotheker AG, Vienna, Austria). Staining with Alcian Blue (Chroma, Waldeck GmbH, Münster, Nordrhein-Westfalen, Germany) to confirm chondrogenic differentiation, von Kossa staining (Silver nitrate, Merck, Darmstadt, Hessen, Deutschland; Natriumthiosulfat; Fluka, Sigma Aldrich, St. Louis, MO, USA) to confirm

osteogenic differentiation and Oil Red O (Sigma-Aldrich, St. Louis, MO, USA) to confirm adipogenic differentiation was performed .

2 Cell characterization: Results

FACS analysis confirmed positive expression of CD29, CD44 and CD166 and a lacking expression of CD31 and CD45 (suppl. fig. S4). Isotype controls (IgG1 and IgG2a) were negative. FACS histograms are depicted in suppl. fig. 1. In addition, mass spectrometry confirmed positive expression of CD44, CD73, CD90 and a lacking expression of CD34 and CD45 (FDR<0.01 for both protein and peptide levels, at least two peptides per protein). Umbilical cord blood derived MSCs showed trilineage (chondrogenic, osteogenic and adipogenic) differentiation following culture in the corresponding differentiation medium for 21 days.
