# Supplementary figures and images for: Fetal Immunomodulatory Environment Following Cartilage Injury—The Key to CARTILAGE Regeneration?
Source: Int J Mol Sci. 2021 Nov 30;22(23):12969. doi: 10.3390/ijms222312969 (PMC8657887; doi:10.3390/ijms222312969)

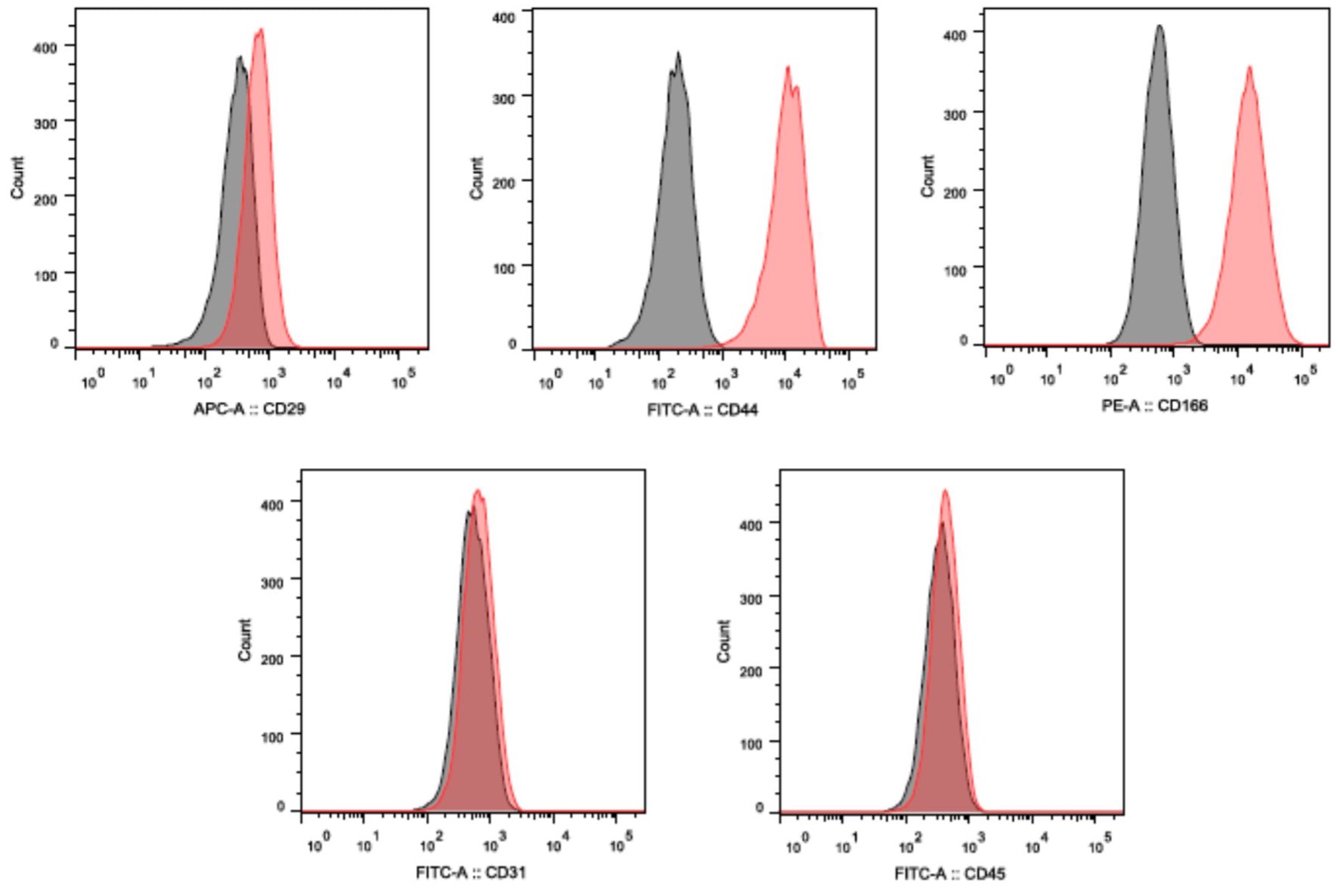

Supplement: Supplementary file 1 [file ijms-22-12969-s001.zip › S4_Suppl_fig.jpg]
